# 4066 In Teenagers Who Smoke Cigarettes Are Nicotine Patches as Compared to Placebo Effective to Decrease the Number of Cigarettes Smoked? – A Systematic Review

**DOI:** 10.1017/cts.2020.408

**Published:** 2020-07-29

**Authors:** Obumneke A Amadi

**Affiliations:** 1 George Washington University

## Abstract

OBJECTIVES/GOALS: The aim was to examine whether nicotine patch was more effective in encouraging abstinence from cigarettes smoking compared to placebo. METHODS/STUDY POPULATION: Randomized controlled trials involving the general teenage age group smokers who were current smokers-“smoked less than 100 cigarettes over their lifetime and smoked at the time of the interview. Databases were searched for relevant studies reported in English that employed a randomized design published since 2000. Two authors extracted data and assessed quality. The primary outcomes and prioritization were continuous abstinence at 3, 6 and 12-month follow-up or more for the number of patients who responded to treatment, defined as a reduction/abstinence. Heterogeneity between studies did not preclude combined analyses of the data. RESULTS/ANTICIPATED RESULTS: 4 of 266 publications were included. Four studies reported positive effects on smoking cessation at end of treatment: (1) nicotine patches improved continuous abstinence at 6 weeks – 9 weeks months; (2) nicotine patch improved continuous abstinence at 3 to 6 months; (3) nicotine patches improved continuous abstinence 6 and 12 months; (4) nicotine patches improved continuous abstinence at 6 months – 12 and 24 months (5). All studies showed, continuous abstinence at follow up differed in percentage between groups both at 6 weeks through 24 months, with NRT (Nicotine patch) intervention groups achieving higher rates in most of the studies compared to placebo intervention group. Conclusions: NRT intervention methods seem to increase smoking abstinence in those treated for smoking cessation. Further and larger sample size studies are required to make stronger the base of evidence. DISCUSSION/SIGNIFICANCE OF IMPACT: Four randomized controlled trials investigating the effectiveness of smoking cessation interventions, for teenagers who smoke cigarettes were identified for inclusion in this review. Four of the studies reported significant effects on smoking cessation, providing evidence of effectiveness of NRT (nicotine patch), behavioral support and combinations of the two, although not all trials intervention treatments found an effect. The four studies reported important intervention effects at both the short and long follow-ups required: 6 weeks up to the 24 months, thereby, providing stronger evidence to support the effectiveness of NRT intervention on smoking cessation. All studies showed some evidence of improved smoking abstinence outcomes. The four studies had in common that the smoking cessation interventions provided a combination of intent to treat prevention, and of all the clinical trials none of them suggested a negative effect of smoking cessation treatment on substance use outcomes using NRT. However, the studies used reliable methods and reported their cases properly, but the small number of studies reviewed for the systematic review makes the conclusion about the effectiveness of these interventions uncertain. The papers visibly stated how the trials protected against bias, as indicated by the Yes (low risk). No (high risk) and U as “unclear risk.” All four studies conducted a random sequence generation of participants enrolled into the study sample.

